# An alternative approach for estimating the number needed to treat for survival endpoints

**DOI:** 10.1371/journal.pone.0223301

**Published:** 2019-10-18

**Authors:** Zhao Yang, Guosheng Yin

**Affiliations:** 1 Department of Statistics and Actuarial Science, The University of Hong Kong, Hong Kong SAR, China; 2 Department of Biostatistics, The University of Texas MD Anderson Cancer Center, Houston, Texas, United States of America; Roswell Park Cancer Institute, UNITED STATES

## Abstract

To investigate the issues of the NNT based on the absolute risk reduction (ARR), namely NNT_ARR_; and to propose an alternative definition and an estimation procedure based on the restricted mean survival time (RMST), namely NNT_RMST_, for RCTs. Three recent clinical trials with survival endpoints, representing different scenarios, were selected to compare the performance of the NNT_ARR_ and NNT_RMST_. For each trial, both versions of NNT were estimated using the reconstructed individual-level data, and the average life gain (ALG) was derived to show the differences between the NNT_ARR_ and NNT_RMST_. Four hypothetical scenarios were constructed to further explore the advantages and disadvantages of each definition of the NNT for survival endpoints. For the illustrative trial examples, the NNT_ARR_ failed to capture the profile of the treatment effect over time as it is calculated at a specific time point. Sometimes it may even result in misinterpretations of the treatment benefit. In particular, when either the observed event rates are low, the two survival curves cross, or a mixture of survival patterns exist. In contrast, the NNT_RMST_ based on the average survival (or event-free) time can quantify the treatment effect more accurately and its interpretation is more intuitive and clinically meaningful. The NNT_RMST_ can be used as an alternative measure for quantifying treatment effect in RCTs, especially so in the case of the ALG, which helps practitioners to better understand the magnitude of the benefit conferred by treatment.

## Introduction

Well-designed and properly executed randomized controlled trials (RCTs) provide the best evidence for the efficacy of health care interventions or new treatments. It is desirable to construct a single measure that can adequately summarize the treatment benefit and be easily conveyed to patients and clinicians.[1] The number needed to treat (NNT) is a popular and intuitive measure in RCTs to quantify the magnitude of the treatment effect.[2] For survival endpoints, the NNT (or NNT_ARR_) is computed as the reciprocal of the absolute risk reduction (ARR) between the treatment and the control group, which is the difference in the Kaplan-Meier (KM) estimated survival rates or the difference in the cumulative incidences at a time point of clinical interest (see S1 Appendix).[3, 4] For the past three decades, the NNT has been widely advocated by medical journals[5, 6] as well as the Cochrane[7] and the Consolidated Standards of Reporting Trials (CONSORT)[8, 9] groups, because it is more transparent to express the magnitude of the treatment effect using the number of patients needed to treat in order to prevent one additional adverse event during a specific follow-up period.

Despite its many advantages,[5, 6, 10, 11] the NNT_ARR_ has been criticized for certain poor statistical properties.[12, 13] In particular, when either the observed event rates for both groups are low, the survival curves cross, or a mixture of survival patterns exist. In these situations, the NNT_ARR_ may fail to capture the profile of the treatment effect over time, thus leading to misinterpretations of the benefit conferred by treatment to some extent. For example, when the two survival curves are close or cross at a chosen time *t* of clinical interest, the corresponding difference in the KM estimates would be close to zero or even becomes a negative value, which results in either a very large or a negative value of the NNT_ARR_. Moreover, the calculation of the NNT_ARR_ depends on the truncated binary endpoints by ignoring the entire process of events and censoring during the *t*-period follow-up, which thus neglects some critical information, resulting in a methodology which cannot reflect the average survival (or event-free) time of patients for both groups. In addition, several other methods have been developed to compute NNT, such as the pseudo-value-based method,[14] the hazard-ratio-based method, [3] and the risk-difference-based method. [15] However, various assumptions are required for these methods. For example, the hazard-ratio-based method requires the validation of the proportional hazard assumption between two groups, which results in an inaccurate NNT when the proportional hazards assumption is not satisfied. [16]

Recently, the restricted mean survival time (RMST), corresponding to the average survival time of patients being followed up to a specific time point, has been advocated to quantify the treatment effect.[17–19] The RMST is typically measured by the area under the KM curve or the area above the cumulative incidence curve from 0 to a specific time point. To better quantify NNT for survival endpoints, we propose the RMST-based NNT as an alternative measure for the NNT_ARR_. We use the reconstructed survival data based on the algorithm by Guyot et al.[20] from three recent clinical trials to illustrate the limitations of the NNT_ARR_ and discuss the advantages and disadvantages of the NNT_RMST_. A general guideline for calculating and reporting the NNT_RMST_ is then provided to facilitate its use in clinical practice.

## Materials and methods

### Number needed to treat based on the restricted mean survival time (NNT_RMST_)

As an alternative to the NNT_ARR_, the NNT_RMST_ provides an intuitive measure to quantify the number of patients needed to treat in order to gain the observed difference in mean survival time for a death or an event (see S1 Appendix). The difference in RMSTs represents the average gain in survival time for patients receiving the experimental treatment in comparison with the control during the *t*-period follow-up.[21] However, the mean survival time for a death or an event is often unknown in clinical practice, and thus we convert it to be the RMST of patients in the control group. The NNT_RMST_ is defined as the RMST in the control group divided by the difference in RMSTs between the two groups up to a chosen time *t*. The NNT_RMST_ can be interpreted as follows.

(1) If the RMST in the treatment group is larger than that in the control group at the chosen time *t*, the NNT_RMST_ is the number of patients needed in the treatment to prevent an extra death or an event in comparison with the control during the *t*-period follow-up.

(2) If the RMST in the treatment group is smaller than that in the control group, a negative NNT_RMST_ is obtained, which conveys a poorer outcome for the treatment. The NNT_RMST_ should then be interpreted as the number needed to treat to harm (NNTH), i.e., the number of patients needed in the treatment group to cause an extra death or an event in comparison with the control during the *t*-period follow-up.

To quantify the uncertainty of the NNT_RMST_, we construct its confidence interval (CI) by inverting the lower and upper boundaries of the ratio of RMSTs between the two groups minus 1 (See S1 Appendix for more details). For example, suppose the RMST for the control group is 1.0 year, the difference in RMSTs is 0.1 and the 95% CI for the ratio of RMSTs is 0.95 to 1.2 during 1-year follow-up. The point estimate of NNT_RMST_ is 10 with the corresponding 95% CI of -20 to 5. In such cases, the 95% CI of the NNT_RMST_ should be interpreted as the number needed to benefit (NNT_RMST_), which is from 5 to ∞, and the number needed to treat to harm (NNT_RMST_), which is from 20 to ∞. In more concise notation, it can be termed as NNT_RMST_ = 10 (NNT_RMST_ 5 to ∞ to NNT_RMST_ 20).

### Average life gain (ALG)

Typically, there is no criterion to make a direct comparison between the NNT_ARR_ and NNT_RMST_. Toward this goal, we introduce a new concept of the average life gain, which is defined as the additional average survival time of patients receiving the treatment in comparison with the control group during the *t*-period follow-up. For the NNT_RMST_, the ALG_RMST_ is the difference in RMSTs between the treatment and the control group up to time *t*. For the NNT_ARR_, the ALG_ARR_ is computed as the mean survival time for one death or an event in patients up to time *t* (which can be approximated as the RMST in the control group) divided by the NNT_ARR_.

## Results and discussion

### Example 1. Radical prostatectomy trial

**Fig 1:**
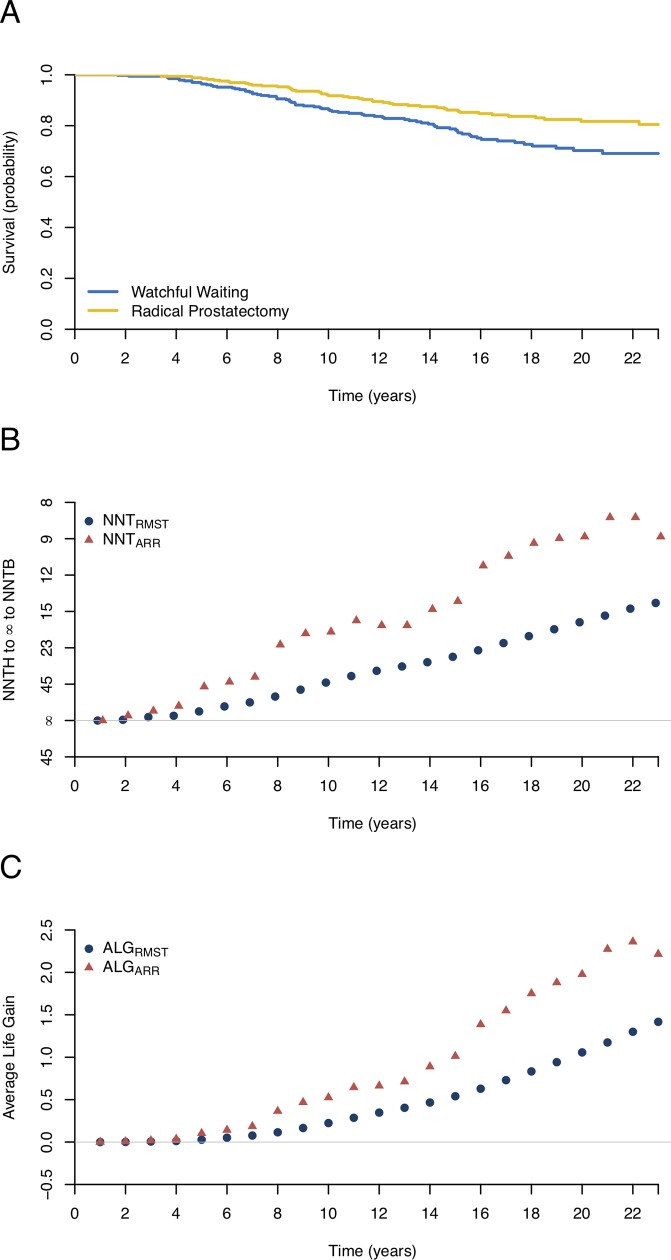
Estimated survival curves, numbers needed to treat (NNTs), and the average life gain (ALG) based on the reconstructed data from the radical prostatectomy study. (A) Kaplan-Meier curves for the radical prostatectomy and watchful waiting groups. (B) The NNTs based on the difference in restricted mean survival times (NNT_RMST_) or the absolute risk reduction (NNT_ARR_). The rescaled y-axis accommodating infinity to distinguish NNT to harm (NNTH) and NNT to benefit (NNTB). (C) The ALG for the NNT_ARR_ and NNT_RMST_ during the follow-up time.

The SPCG-4 (Scandinavian Prostate Cancer Group Study Number 4)[22] trial tested whether radical prostatectomy would reduce the mortality among men with localized prostate cancer in comparison with watchful waiting. A total of 695 patients were randomized to the radical prostatectomy or the watchful waiting group from October 1989 to February 1999 and followed until December 2017. The primary endpoint was death from prostate cancer with death from other causes treated as a competing event. Fig 1A shows the survival curves, where no significant treatment effect is observed during the first four years, and afterwards the survival curves appear to be notably different.

In addition to the cumulative incidences and the hazard ratios, Bill-Axelson et al.[22] also reported the absolute difference in risk and the corresponding NNT to quantify the treatment effect at the 23-year follow-up. The estimated NNT_ARR_ was 8.8 (95% CI, 5.2 to 27.8), which indicated that the number of patients needed in the radical prostatectomy group to prevent one death was 8.8 during the 23-year follow-up. Clinically, the NNT_ARR_ reflects the cumulative treatment effect at the 23-year follow-up rather than the profile of the benefit conferred by the radical prostatectomy over time as the KM curves are initially close, and then diverge after 4 years (Fig 1A). Furthermore, the estimated NNT_ARR_ at 20 and 23 years were exactly the same with the value of 8.8, while the average survival times for patients being followed up to 20 and 23 years were different (Table 1). In such cases, the NNT_ARR_ estimated at an arbitrary time point may not adequately account for the long-term follow-up effect. As a result, it is rather difficult to explain the equality of the NNT_ARR_s at 20 and 23 years to clinicians and patients. Last but not least, the uncertainty of the estimated NNT_ARR_ depends on the CI of the ARR at the specific time point, which further relies on the event rates in the treatment and control groups, but not on patient exposure time. In some situations, it may also lead to an unstable CI of the NNT_ARR_, particularly when the observed event rates are low during the long-term follow-up period (Table 1).

**Table 1:** Comparison of the NNT_ARR_ and NNT_RMST_ based on three real trials examples.

Time	NNT_ARR_ (95% CI)	NNT_RMST_ (95% CI)	Mean survival time for one death up to time 𝑡 (95% CI)
**Radical Prostatectomy Trial (years)**			
5	47.6 (22.2 to ∞ to 333.3)	176.8 (83.3 to ∞ to 1000.0)	5.0 (4.9 to 5.0)
10	18.2 (9.6 to 166.7)	42.5 (23.3 to 250.0)	9.5 (9.4 to 9.7)
15	13.5 (7.3 to 90.9)	25.3 (14.1 to 111.1)	13.7 (13.4 to 14.0)
20	8.8 (5.2 to 27.8)	16.4 (9.6 to 52.6)	17.4 (16.8 to 17.9)
23	8.8 (5.1 to 33.3)	13.7 (8.1 to 40.0)	19.4 (18.7 to 20.1)
**Atezolizumab and Nab-Paclitaxel Trial (months)**			
6	10.1 (6.0 to 38.5)	23.9 (14.6 to 63.8)	4.5 (4.3 to 4.6)
12	15.6 (8.3 to 142.9)	8.4 (5.3 to 19.9)	6.3 (5.9 to 6.6)
15	29.4 (11.4 to ∞ to 50.0)	6.7 (4.1 to 17.3)	6.7 (6.3 to 7.2)
21	29.4 (11.2 to ∞ to 47.6)	6.0 (3.6 to 18.3)	7.3 (6.7 to 7.9)
24	55.6 (13.3 to ∞ to 25.6)	5.6 (3.3 to 17.5)	7.4 (6.8 to 8.1)
**Caplacizumab Trial (days)**			
1	16.9 (7.0 to ∞ to 40)	82.4 (32.7 to ∞ to 140.2)	1.0 (1.0 to 1.0)
3	4.6 (2.7 to 15.9)	13.4 (5.9 to ∞ to 49.3)	2.4 (2.2 to 2.5)
12	23.8 (7.0 to ∞ to 17.2)	2.3 (1.3 to 10.5)	3.1 (3.5 to 3.7)
16	23.8 (7.0 to ∞ to 17.2)	2.2 (1.2 to 26.9)	3.3 (2.5 to 4.1)
20	-52.6 (14.9 to ∞ to 9.5)	2.3 (1.1 to ∞ to 25.4)	3.5 (2.4 to 4.6)

An alternative approach to summarizing the effect of the radical prostatectomy is based on the NNT_RMST_. The estimated NNT_RMST_ decreased with the follow-up time, which qualitatively reflected the benefit of the radical prostatectomy. These trends were identical to those in the NNT_ARR_, confirming the advantage of the NNT_RMST_ in capturing the necessary information. This is why the NNT_RMST_ and NNT_ARR_ curves diverge in Fig 1B. Moreover, the NNT_RMST_s at 20 and 23 years were 16.4 (95% CI, 9.6 to 52.6) and 13.7 (95% CI, 8.1 to 40.0) respectively, which suggested that the number needed to treat at 23 years was smaller than that at 20 years owing to the cumulative effect conferred by the radical prostatectomy. In such cases, the NNT_RMST_ provides a more reasonable measure than the NNT_ARR_. To further explore the difference between the NNT_ARR_ and NNT_RMST_, Fig 1C presents the ALG respectively based on the NNT_RMST_ and NNT_ARR_ during the follow-up period. It is evident that the ALG based on the NNT_ARR_ was overestimated compared with that based on the NNT_RMST_, leading to an exaggerated treatment effect. Clearly, the NNT_RMST_ inherits the nature of the survival time and provides a more accurate and intuitive estimate than the NNT_ARR_.

### Example 2. Atezolizumab and nab-paclitaxel trial

**Fig 2:**
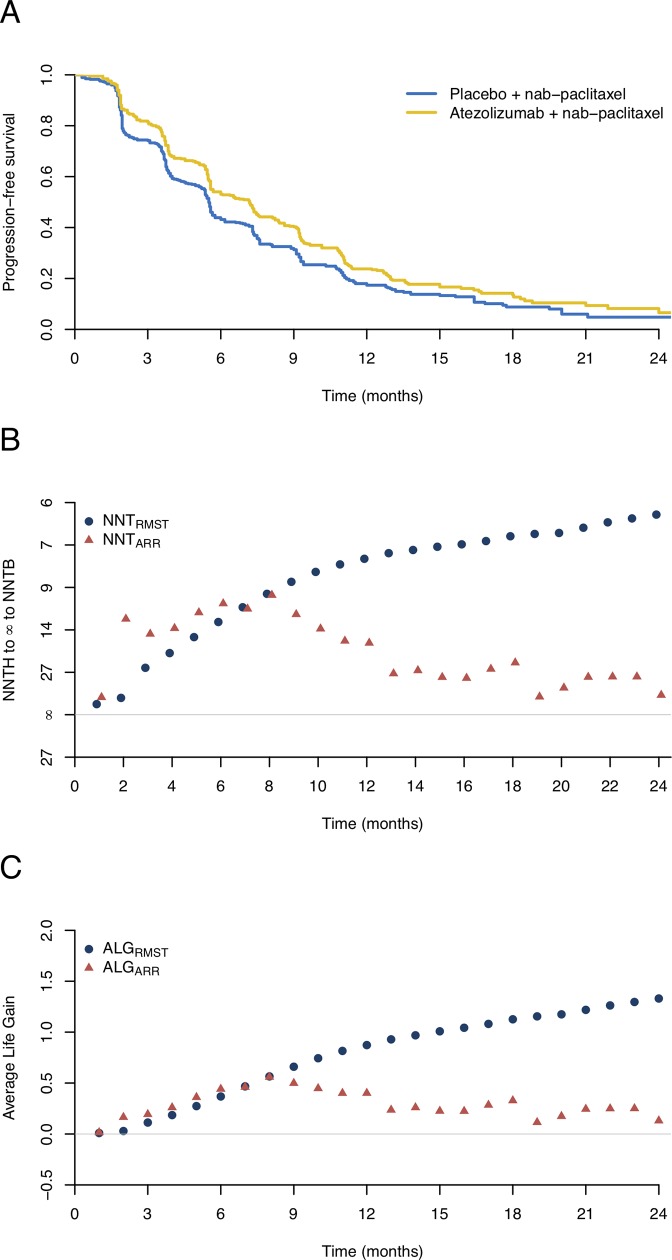
Estimated survival curves, numbers needed to treat (NNTs), and the average life gain (ALG) based on the reconstructed data from the atezolizumab and nab-paclitaxel trial. (A) Kaplan-Meier curves for the atezolizumab plus nab-paclitaxel and the placebo plus nab-paclitaxel groups. (B) The NNTs based on the difference in restricted mean survival times (NNT_RMST_) or the absolute risk reduction (NNT_ARR_). The rescaled y-axis accommodating infinity to distinguish NNT to harm (NNTH) and NNT to benefit (NNTB). (C) The ALG for the NNT_ARR_ and NNT_RMST_ during the follow-up time.

The IMpassion130 trial,[23] a phase 3 trial of an anti-PD-L1 or anti-PD-1 antibody in patients with metastatic triple-negative breast cancer, was conducted to evaluate the potential benefit of first-line atezolizumab plus nab-paclitaxel (AP) in comparison with placebo plus nab-paclitaxel (PP). The trial enrolled 452 patients in each group with a median follow-up of 12.9 months. The primary endpoint was progression-free survival (PFS) as shown in Fig 2A. Although the survival curves are initially close, they begin to diverge and then move close again multiple times during the follow-up period. The survival curve of the AP group always stays above that of the PP group, suggesting that patients receiving AP had improved PFS. We estimated the NNT_ARR_ and its 95% CI at different time points. The NNT_ARR_ was 10.1 (95% CI, 6.0 to 38.5) at 6 months and then continuously increased with the follow-up time, as shown in Table 1 and Fig 2B. It is interesting to note that such NNT_ARR_s only capture the cumulative treatment effect at a specific time point, and may be misinterpreted in clinical practice. For example, the NNT_ARR_s were 29.4 (95% CI, 11.2 to 47.6) and 55.6 (95% CI, 13.3 to ∞ to 25.6) at 21 and 24 months respectively, which suggests that the number of patients receiving AP to prevent one death at 24 months was 1.9 times higher than that at 21 months. However, the average PFS time for patients being followed up to 21 and 24 months were quite similar with 7.3 and 7.5 months, respectively (Table 1). Such inconsistent results may cause confusion among patients and clinicians.

Moreover, the ALG based on the NNT_ARR_ decreased, particularly at the end of the follow-up period (Fig 2C), which failed to depict the cumulative treatment effect of AP. In contrast, the estimated NNT_RMST_ decreased with the follow-up time, indicating that patients receiving AP had improved PFS. For example, the NNT_RMST_s at 21 and 24 months were 6.0 (95% CI, 3.6 to 18.3) and 5.6 (95%CI, 3.3 to 17.5) with the corresponding ALG of 1.2 and 1.3 months. These results further confirm that the NNT_RMST_ is a more rational choice than the NNT_ARR_ when summarizing the study results and communicating with practitioners in clinical practice.

### Example 3. Caplacizumab trial

**Fig 3:**
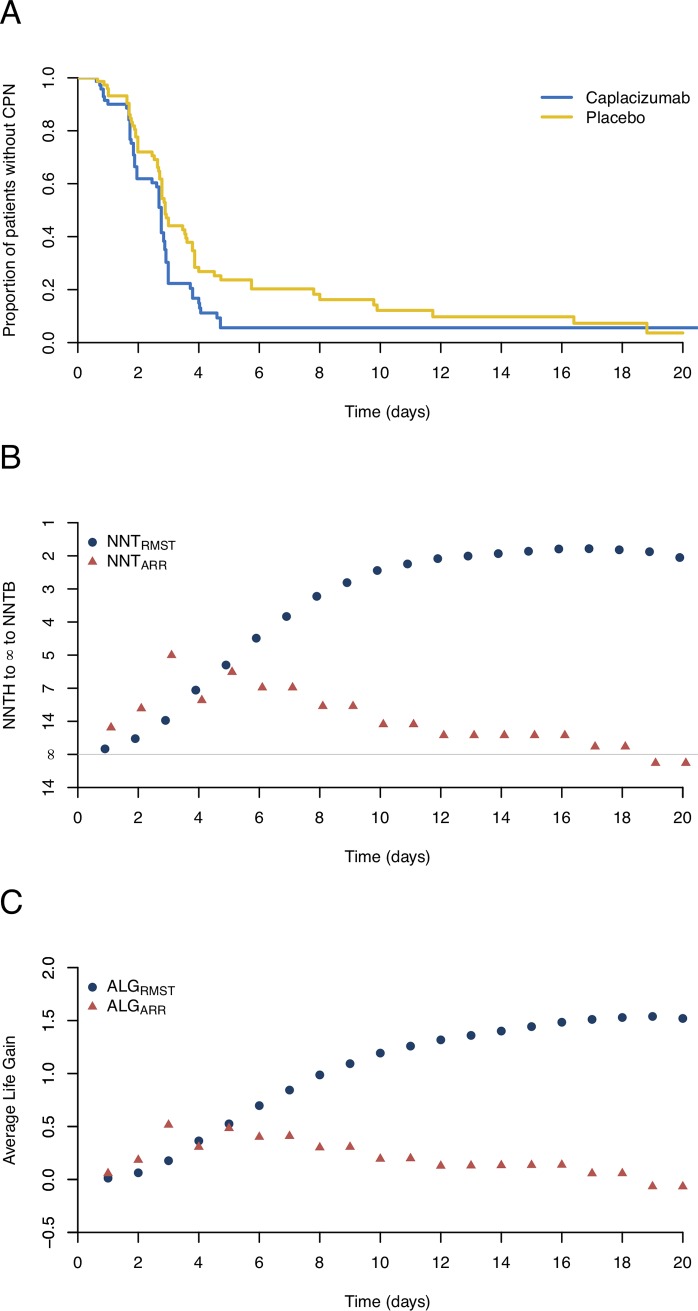
Estimated survival curves, numbers needed to treat (NNTs), and the average life gain (ALG) based on the reconstructed data from the caplacizumab trial. (A) Kaplan-Meier curves for the caplacizumab and placebo groups. (B) The NNTs based on the difference in restricted mean survival times (NNT_RMST_) or the absolute risk reduction (NNT_ARR_). The rescaled y-axis accommodating infinity to distinguish NNT to harm (NNTH) and NNT to benefit (NNTB). (C) The ALG for the NNT_ARR_ and NNT_RMST_ during the follow-up time.

The third example is from a recent clinical trial reported by Scully et al.[24] with an aim to test whether the treatment with caplacizumab could expedite the confirmed normalization of the platelet count in comparison with placebo among patients with acquired thrombotic thrombocytopenic purpura (TTP). A total of 145 patients were randomly assigned to the caplacizumab or placebo to record the time to normalization of the platelet count. The median time to normalization of the platelet count was shorter with caplacizumab (2.7 days; 95% CI, 1.9 to 2.8) than that with placebo (2.9 days; 95% CI, 2.7 to 3.6). Fig 3A shows the proportion of patients without confirmed platelet normalization for the two groups during the follow-up period. The two survival curves are initially indistinguishable, and then diverge and converge twice during the follow-up, before eventually crossing 18 days afterwards. However, the benefit of using caplacizumab continuously exists until the end of the trial, as the average time to confirmed platelet normalization in the caplacizumab group was shorter than that in the placebo group.

The estimated NNT_ARR_ fluctuated with an unstable 95% CI during the follow-up period. For example, the NNT_ARR_s were 4.6 (95% CI, 2.7 to 15.9) and -52.6 (95% CI, 14.9 to ∞ to 9.5) at 3 and 20 days respectively, indicating that the treatment effect of caplacizumab reversed around day 20 (Fig 3A and 3B). In addition, we estimated the NNT_RMST_ at 3 and 20 days to be 13.4 (95% CI, 5.9 to ∞ to 49.5) and 2.3 (95% CI, 1.1 to ∞ to 25.4) with the corresponding ALG of 0.2 and 1.5 days. The results suggested that caplacizumab decreased the time to confirmed platelet normalization, although no significant difference was observed between caplacizumab and placebo, as shown in Table 1 and Fig 3C. In such cases, the NNT_RMST_ outperforms the NNT_ARR_ and provides a more sensible and accurate measure of the treatment effect.

### Hypothetical example

**Fig 4:**
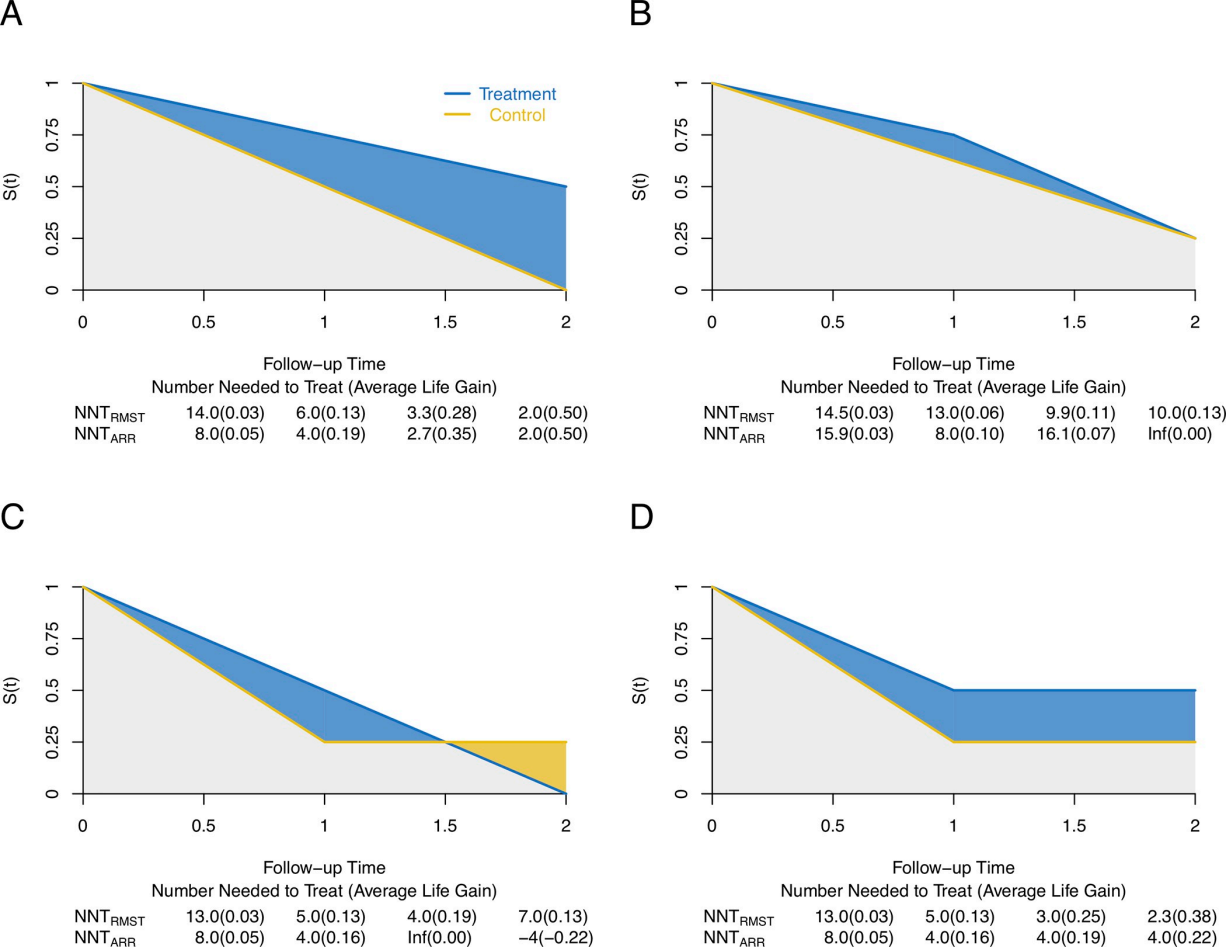
Hypothetical examples representing four different scenarios. (A) Scenario 1: Survival curves with an increasing treatment effect over time. (B) Scenario 2: Survival curves converge at the end of the follow-up. (C) Scenario 3: Survival curves cross during the follow-up period. (D) Scenario 4: Survival curves with a mixture of short- and long- term effects. The area up to time *t* under each scenario between treatment and control groups represent the average life gain (ALG).

To further explore the advantages and disadvantages of each definition of the NNT for survival endpoints, four hypothetical scenarios were constructed. Scenario 1 reflects two survival curves with an increasing treatment effect over time (Fig 4A). Both NNT_RMST_ and NNT_ARR_ decrease with the follow-up time, and the corresponding ALG increases. Compared with the NNT_RMST_, the NNT_ARR_ may overestimate the treatment effect during the follow-up period as it does not account for patient exposure time. In Scenario 2, two survival curves converge at the end of the follow-up (Fig 4B). The survival curve in the treatment group always stays above that in the control group until the end of the study, which indicates that patients receiving the experimental treatment have prolonged survival time. The ALG_RMST_ increases with the follow-up time, whereas the ALG_ARR_ first increases, but then decreases to 0. Scenario 3 reflects that two survival curves cross during the follow-up period (Fig 4C). The overall (or cumulative) treatment effect still exists, as the mean survival time in the treatment group is larger than that in the control group. The NNT_ARR_ captures the local treatment effect rather than the overall treatment effect, e.g., NNT_ARR_ is infinity at time 1.5. In contrast, the NNT_RMST_ depicts the cumulative treatment effect at a chosen time point and reflects the profile of the treatment effect over the follow-up time. In Scenario 4, two survival curves with a mixture of short- and long- term effects are presented (Fig 4D). It is evident that the treatment effect continuously exists during the follow-up period. However, the NNT_ARR_ is the same of 4 from time 1 to 2, which fails to reflect the treatment benefit and may further cause misunderstanding. In contrast, the NNT_RMST_ provides a more reasonable measure as it decreases gradually until the end of the study.

**Fig 5:**
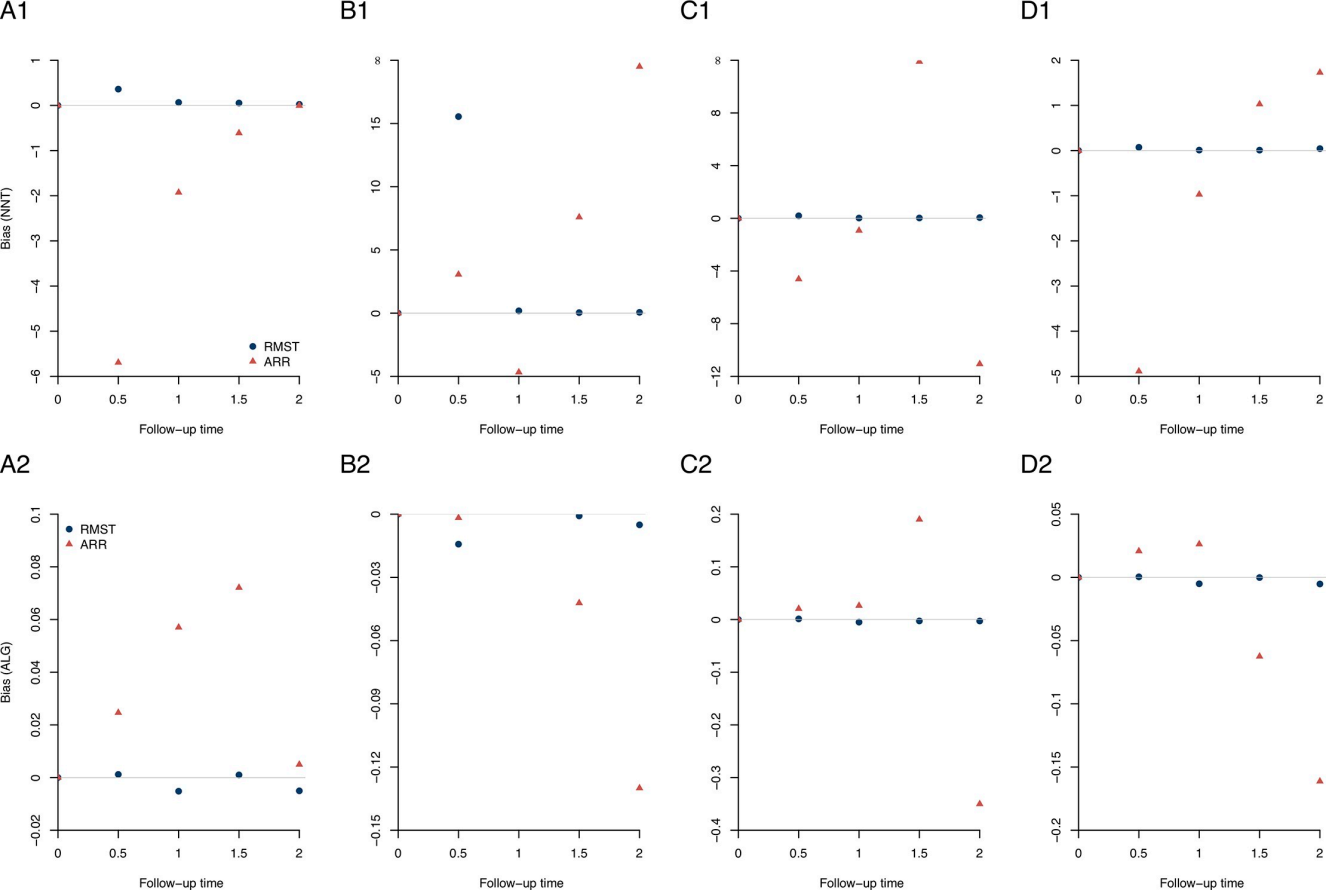
Simulation results of NNT_RMST_ and NNT_ARR_ under four hypothetical scenarios. The upper panel presents the bias of NNT at different time points, and the lower panel shows the bias of the ALG at different time points. (A1-2) Scenario 1: Survival curves with an increasing treatment effect over time; (B1-2) Scenario 2: Survival curves converge at the end of the follow-up; (C1-2) Scenario 3: Survival curves cross during the follow-up period; (D1-2) Scenario 4: Survival curves with a mixture of short- and long- term effects.

In addition, we conducted simulation studies under the four hypothetical scenarios to examine the performance of NNT_RMST_ and NNT_ARR_ in terms of the biases of NNT and ALG. A total of 5000 simulation studies with a sample size of 500 were carried out, and the simulation results in Fig 5 show that the NNT_RMST_ outperforms the NNT_ARR_, as the NNT_RMST_ inherits the advantages of quantifying the average “survival” (event-free) time. It is worth noting that the performance of NNT_RMST_ is worse only under scenario 2 at time point *t* = 0.5, because the follow-up time is short and there is little information and thus large variation in RMST by *t* = 0.5.

## Discussion

As an essential component of RCTs, interpreting the evidence of the treatment effect to practitioners plays a vital role in their decision making under the risk-benefit consideration. The popularity of the ARR in medical research makes the NNT_ARR_ a primary tool for quantifying treatment effect,[2, 6, 25] although it has significant drawbacks. Nowadays, the RMST-based quantitative measures have been advocated to be a primary tool for clinical trials and to help practitioners to understand the treatment effect better.[17, 18, 26–29] We mainly compared pros and cons between the NNT_ARR_ and NNT_RMST_ via the ALG along with three real examples and four hypothetical scenarios. The NNT_RMST_ can accurately convey the likelihood of the treatment success and aligns more closely with the patient perspective by converting the treatment effect into “the chance of benefiting 1 in X”, which further facilitates the rational decision making when several treatments are available. In addition, the uncertainty of the benefit is quantified by constructing the CI of the NNT_RMST_. Not only does the NNT_RMST_ inherit the intuitive interpretation of the NNT_ARR_ but it also overcomes the shortcomings of the NNT_ARR_ to some extent, providing a better alternative measure. For example, the NNT_RMST_ (1) provides an easy-to-interpret clinically meaningful summary of the treatment effect; (2) has a well-established calculation procedure; (3) conveys the uncertainty of the NNT_RMST_ at a specific time point *t*; (4) reflects the profile of the treatment effect during the follow-up period, which further explains why the NNT_RMST_ and NNT_ARR_ curves diverge in the figures; (5) has a coherent estimate when either the event rates are low, the survival curves cross, or a mixture of survival patterns exist; (6) makes full use of the available information and accounts for the follow-up effect; (7) quantifies the average “survival” (event-free) time directly.

Nevertheless, it is arguable that determination of the more appropriate measure depends on the real situation rather than either the estimation procedure or the stability of the method throughout the entire follow-up period. For example, if survival to a specific time is a critical indicator of treatment effectiveness, then the NNT_ARR_ at the specific time may be more appropriate. Furthermore, there still exist some limitations of the NNT_RMST_. For instance, the NNT_RMST_ cannot reflect the importance of endpoints, fails to convey the cost-effectiveness of the treatment, and requires a prespecified follow-up time. Despite these limitations, the NNT_RMST_ is still of great value as it reflects the treatment effect between the two groups accurately and intuitively. We have developed the R software package “nnt” to facilitate the calculation of the NNT_RMST_, which can be freely downloaded from R-CRAN.

## Conclusion

The NNT_RMST_T can be used as an alternative measure for quantifying treatment effect in RCTs, especially so in the case of the ALG, which helps practitioners to better understand the magnitude of the benefit conferred by treatment.

## Supporting information

S1 Appendix Technical notes. (DOCX)

## References

[1] Guyatt GH, Sackett DL, Sinclair JC, Hayward R, Cook DJ, Cook RJ. Users' guides to the medical literature. IX. A method for grading health care recommendations. Evidence-Based Medicine Working Group. JAMA. 1995;274(22):1800–4. 10.1001/jama.274.22.1800 7500513

[2] Laupacis A, Sackett DL, Roberts RS. An assessment of clinically useful measures of the consequences of treatment. N Engl J Med. 1988;318(26):1728–33. 10.1056/NEJM198806303182605 3374545

[3] Altman DG, Andersen PK. Calculating the number needed to treat for trials where the outcome is time to an event. BMJ. 1999;319(7223):1492–5. 10.1136/bmj.319.7223.1492 10582940 PMC1117211

[4] Altman DG. Confidence intervals for the number needed to treat. BMJ. 1998;317(7168):1309–12. 10.1136/bmj.317.7168.1309 9804726 PMC1114210

[5] Nuovo J, Melnikow J, Chang D. Reporting number needed to treat and absolute risk reduction in randomized controlled trials. JAMA. 2002;287(21):2813–4. 10.1001/jama.287.21.2813 12038920

[6] Saver JL, Lewis RJ. Number needed to treat: Conveying the likelihood of a therapeutic effect. JAMA. 2019; 321(8):798–799. 10.1001/jama.2018.21971 30730545

[7] Higgins JPT, Green S. Cochrane handbook for systematic reviews of interventions. 2008.

[8] Altman DG, Schulz KF, Moher D, Egger M, Davidoff F, Elbourne D, et al The revised CONSORT statement for reporting randomized trials: Explanation and elaboration. Ann Intern Med. 2001;134(8):663–94. 10.7326/0003-4819-134-8-200104170-00012 11304107

[9] Moher D, Hopewell S, Schulz KF, Montori V, Gotzsche PC, Devereaux PJ, et al CONSORT 2010 explanation and elaboration: Updated guidelines for reporting parallel group randomised trials. BMJ. 2010;340:c869 10.1136/bmj.c869 20332511 PMC2844943

[10] Osiri M, Suarez-Almazor ME, Wells GA, Robinson V, Tugwell P. Number needed to treat (NNT): Implication in rheumatology clinical practice. Ann Rheum Dis. 2003;62(4):316–21. 10.1136/ard.62.4.316 12634229 PMC1754501

[11] Hutton JL. Number needed to treat: Properties and problems. J R Statl Soc Ser A Stat Soc. 2000;163(3):381–402.

[12] Hutton JL. Number needed to treat and number needed to harm are not the best way to report and assess the results of randomised clinical trials. Br J Haematol. 2009;146(1):27–30. 10.1111/j.1365-2141.2009.07707.x 19438480

[13] Suissa D, Brassard P, Smiechowski B, Suissa S. Number needed to treat is incorrect without proper time-related considerations. J Clin Epidemiol. 2012;65(1):42–6. 10.1016/j.jclinepi.2011.04.009 21816576

[14] Andersen PK, Perme MP. Pseudo-observations in survival analysis. Stat Methods Med Res. 2010;19(1):71–99. 10.1177/0962280209105020 19654170

[15] Austin PC. Absolute risk reductions and numbers needed to treat can be obtained from adjusted survival models for time-to-event outcomes. J Clin Epidemiol. 2010;63(1):46–55. 10.1016/j.jclinepi.2009.03.012 19595575

[16] Bowry SK, Schoder V, Apel C. An inadvertent but explicable error in calculating number needed to treat for reporting survival data. J Am Soc Nephrol. 2014;25(5):875–6. 10.1681/ASN.2014020188 24722435 PMC4005325

[17] Uno H, Claggett B, Tian L, Inoue E, Gallo P, Miyata T, et al Moving beyond the hazard ratio in quantifying the between-group difference in survival analysis. J Clin Oncol. 2014;32(22):2380–5. 10.1200/JCO.2014.55.2208 24982461 PMC4105489

[18] Uno H, Wittes J, Fu H, Solomon SD, Claggett B, Tian L, et al Alternatives to hazard ratios for comparing the efficacy or safety of therapies in noninferiority studies. Ann Intern Med. 2015;163(2):127–34. 10.7326/M14-1741 26054047 PMC4510023

[19] Yin G. *Clinical Trial Design*: *Bayesian and Frequentist Adaptive Methods*: John Wiley & Sons; 2012.

[20] Guyot P, Ades AE, Ouwens MJ, Welton NJ. Enhanced secondary analysis of survival data: Reconstructing the data from published Kaplan-Meier survival curves. BMC Med Res Methodol. 2012;12:9 10.1186/1471-2288-12-9 22297116 PMC3313891

[21] Zhao L, Tian L, Uno H, Solomon SD, Pfeffer MA, Schindler JS, et al Utilizing the integrated difference of two survival functions to quantify the treatment contrast for designing, monitoring, and analyzing a comparative clinical study. Clin Trials. 2012;9(5):570–7. 10.1177/1740774512455464 22914867 PMC3705645

[22] Bill-Axelson A, Holmberg L, Garmo H, Taari K, Busch C, Nordling S, et al Radical prostatectomy or watchful waiting in prostate cancer—29-year follow-up. N Engl J Med. 2018;379(24):2319–29. 10.1056/NEJMoa1807801 30575473

[23] Schmid P, Adams S, Rugo HS, Schneeweiss A, Barrios CH, Iwata H, et al Atezolizumab and nab-paclitaxel in advanced triple-negative breast cancer. N Engl J Med. 2018;379(22):2108–21. 10.1056/NEJMoa1809615 30345906

[24] Scully M, Cataland SR, Peyvandi F, Coppo P, Knobl P, Kremer Hovinga JA, et al Caplacizumab treatment for acquired thrombotic thrombocytopenic purpura. N Engl J Med. 2019;380(4):335–46. 10.1056/NEJMoa1806311 30625070

[25] Mendes D, Alves C, Batel-Marques F. Number needed to treat (NNT) in clinical literature: An appraisal. BMC Med. 2017;15(1):112 10.1186/s12916-017-0875-8 28571585 PMC5455127

[26] Trinquart L, Jacot J, Conner SC, Porcher R. Comparison of treatment effects measured by the hazard ratio and by the ratio of restricted mean survival times in oncology randomized controlled trials. J Clin Oncol. 2016;34(15):1813–9. 10.1200/JCO.2015.64.2488 26884584

[27] Pak K, Uno H, Kim DH, Tian L, Kane RC, Takeuchi M, et al Interpretability of cancer clinical trial results using restricted mean survival time as an alternative to the hazard ratio. JAMA Oncol. 2017;3(12):1692–6. 10.1001/jamaoncol.2017.2797 28975263 PMC5824272

[28] Uno H, Claggett B, Tian L, Fu H, Huang B, Kim DH, et al Adding a new analytical procedure with clinical interpretation in the tool box of survival analysis. Ann Oncol. 2018;29(5):1092–4. 10.1093/annonc/mdy109 29617717 PMC5961386

[29] Liang F, Zhang S, Wang Q, Li W. Treatment effects measured by restricted mean survival time in trials of immune checkpoint inhibitors for cancer. Ann Oncol. 2018;29(5):1320–4. 10.1093/annonc/mdy075 29788167

