# Supplementary material for: An alternative approach for estimating the number needed to treat for survival endpoints
Source: PLoS One. 2019 Oct 18;14(10):e0223301. doi: 10.1371/journal.pone.0223301 (PMC6799908; doi:10.1371/journal.pone.0223301)
Supplement: S1 Appendix — (DOCX) [file pone.0223301.s001.docx]

**An Alternative Approach for Estimating the Number Needed to Treat for Survival Endpoints**

Zhao Yang and Guosheng Yin

Contents

[Appendix 1. NNT Based on the Absolute Risk Reduction ($\mathbf{NNT}_{\mathbf{ARR}}$) 2](#_Toc8845173)

[Appendix 2. NNT Based on the Difference in Restricted Mean Survival Time ($\mathbf{NNT}_{\mathbf{RMST}}$) 3](#_Toc8845174)

# Appendix 1. NNT Based on the Absolute Risk Reduction ($\mathbf{NNT}_{\mathbf{ARR}}$)

For RCTs with survival endpoints, the calculation of the $\mathrm{NNT}_{\mathrm{ARR}}$is defined as the reciprocal of the absolute risk reduction (ARR),

$$\mathrm{NNT}_{\mathrm{ARR}}(t) = \frac{1}{ARR(t)}=\frac{1}{S_{E}\left( t \right)-S_{C}\left( t \right)}$$

where $S_{E}(t)$ and $S_{C}(t)$ are respectively the estimated survival rates for the experimental treatment and control groups at a chosen time $t$ of clinical interest. The 95% confidence interval (CI) of the $\mathrm{ARR}\left( t \right)$ can be constructed as

$$\mathrm{ARR}\left( t \right)\pm1.96\times SE\left( \mathrm{ARR}\left( t \right) \right)$$

where

$$SE\left( \mathrm{ARR}\left( t \right) \right)=\sqrt{\mathrm{Var}\left( S_{E}\left( t \right) \right)+\mathrm{Var}(S_{C}\left( t \right))}$$

and ${\mathrm{Var}(S}_{E}(t))$ and ${\mathrm{Var}(S}_{C}(t))$ are the variances of the Kaplan-Meier estimates for the experimental treatment and control groups at time point $t$, respectively. The lower (LCI) and upper (UCI) 95% CI of the $\mathrm{NNT}_{\mathrm{ARR}}$can then be calculated as follows,

$$LCI(\mathrm{NNT}_{\mathrm{ARR}})=\frac{1}{\mathrm{ARR}+1.96\times SE(\mathrm{ARR})}$$

and

$$UCI(\mathrm{NNT}_{\mathrm{ARR}})=\frac{1}{\mathrm{ARR}-1.96\times SE(\mathrm{ARR})}$$

If the 95% CI of the $\mathrm{NNT}_{\mathrm{ARR}}$ covers 0, it can be interpreted as the number needed to treat to benefit (NNTB) and the number needed to treat to harm (NNTH). For example, if the ARR is 10% with a 95% CI of -5% to 20%, then the $\mathrm{NNT}_{\mathrm{ARR}}$ is 10 (95% CI: -20 to 5). As a result, the 95% CI can be represented as 5 to $\infty$ for NNTB, and 20 to $\infty$ for NNTH, which is denoted as 5 to $\infty$ to 20.

# Appendix 2. NNT Based on the Difference in Restricted Mean Survival Time ($\mathbf{NNT}_{\mathbf{RMST}}$)

As an alternative to the absolute risk reduction, the restricted mean survival time (RMST), which is defined as the average survival time for subjects followed up to time $t$, has been advocated to quantify patient survival. The $RMST(t)$ is typically calculated as the area under the Kaplan-Meier survival curve or the area above the cumulative incidence curve from 0 to time point $t$. It can be interpreted as the mean survival time limited to a specific time point $t$, which equals to the area under the survival curve $S(x)$ from $x=0$ to $x=t$,

$$\mathrm{RMST}\left( t \right)=E[\min(T,t)]= \int_{0}^{t} S(x)dx$$

where $S(x)$ can be estimated using the Kaplan-Meier method. The $\mathrm{NNT}_{R\mathrm{MST}}$ is defined as

$$\mathrm{NNT}_{R\mathrm{MST}}(t) =\frac{\mathrm{RMST}_{C}\left( t \right)}{\mathrm{RMST}_{E}\left( t \right)-\mathrm{RMST}_{C}\left( t \right)}=\frac{1}{{\mathrm{RMST}_{E}\left( t \right)}/{\mathrm{RMST}_{C}\left( t \right)}-1}$$

where $\mathrm{RMST}_{E}\left( t \right)$ and $\mathrm{RMST}_{C}\left( t \right)$ are RMSTs in the experimental treatment and control arms up to $t$, respectively.

To obtain the 95% CI of the $\mathrm{NNT}_{R\mathrm{MST}}$(t), we first compute the 95% CI of ${\mathrm{RMST}_{E}\left( t \right)}/{\mathrm{RMST}_{C}\left( t \right)}$, that is,

A$SE\left( \log\left( {\mathrm{RMST}_{E}\left( t \right)}/{\mathrm{RMST}_{C}\left( t \right)} \right) \right)=SE\left( \log\left( \mathrm{RMST}_{E}\left( t \right) \right)-\log\left( \mathrm{RMST}_{C}\left( t \right) \right) \right)$

$$\approx\sqrt{\frac{\mathrm{Var}(\mathrm{RMST}_{E}\left( t \right))}{{\mathrm{RMST}_{E}\left( t \right)}^{2}}+\frac{\mathrm{Var}(\mathrm{RMST}_{C}\left( t \right))}{{\mathrm{RMST}_{C}\left( t \right)}^{2}}}$$

The lower (LCI) and upper (UCI) 95% CI of the ${\mathrm{RMST}_{E}\left( t \right)}/{\mathrm{RMST}_{C}\left( t \right)}$ can be estimated as follows,

$$\mathrm{LCI}\left( {\mathrm{RMST}_{E}\left( t \right)}/{\mathrm{RMST}_{C}\left( t \right)} \right)=e^{{{log(RMST}_{E}\left( t \right)}/{\mathrm{RMST}_{C}\left( t \right))}-1.96\times\mathrm{SE}\left( log({\mathrm{RMST}_{E}\left( t \right)}/{\mathrm{RMST}_{C}\left( t \right)}) \right)}$$

$$\mathrm{UCI}\left( {\mathrm{RMST}_{E}\left( t \right)}/{\mathrm{RMST}_{C}\left( t \right)} \right)=e^{{{log(RMST}_{E}\left( t \right)}/{\mathrm{RMST}_{C}\left( t \right))}+1.96\times\mathrm{SE}\left( log({\mathrm{RMST}_{E}\left( t \right)}/{\mathrm{RMST}_{C}\left( t \right)}) \right)}$$

Then, the lower (LCI) and upper (UCI) 95% CI of the $\mathrm{NNT}_{R\mathrm{MST}}$(t) can be calculated by inverting those of ${\mathrm{RMST}_{E}\left( t \right)}/{\mathrm{RMST}_{C}\left( t \right)}-1$,

$$LCI(\mathrm{NNT}_{R\mathrm{MST}}(t)) =\frac{1}{\mathrm{UCI}\left( {\mathrm{RMST}_{E}\left( t \right)}/{\mathrm{RMST}_{C}\left( t \right)} \right)-1}$$

$$UCI(\mathrm{NNT}_{R\mathrm{MST}}(t))=\frac{1}{\mathrm{LCI}\left( {\mathrm{RMST}_{E}\left( t \right)}/{\mathrm{RMST}_{C}\left( t \right)} \right)-1}$$

Similar to $\mathrm{NNT}_{\mathrm{ARR}}$, the 95% CI of $\mathrm{NNT}_{R\mathrm{MST}}$ can also be termed as NNTB and NNTH when it covers zero.
